# Dendrimer Dynamics: A Review of Analytical Theories and Molecular Simulation Methods

**DOI:** 10.3390/polym12061387

**Published:** 2020-06-20

**Authors:** Fabio Ganazzoli, Giuseppina Raffaini

**Affiliations:** Dipartimento di Chimica, Materiali ed Ingegneria Chimica “G. Natta”, Politecnico di Milano, Piazza Leonardo da Vinci 32, 20131 Milano, Italy; giuseppina.raffaini@polimi.it

**Keywords:** dendrimers, branched polymers, rheology, relaxation times, hydrodynamic interaction, intrinsic viscosity, viscoelastic complex modulus, dynamic structure factor

## Abstract

The theoretical study of dendrimers is reviewed, considering both analytical approaches and molecular simulation methods. We discuss the effect of molecular symmetry on the degeneracy of the relaxation times, and then the calculation of observable quantities, in particular the intrinsic viscosity, and then the viscoelastic complex modulus and the dynamic structure factor, in comparison with the available experimental data. In particular, the maximum intrinsic viscosity with increasing molar mass is analyzed in some detail. The approximations and/or assumptions of the adopted methods are also described in connection with analogous results for polymer of a different topology, in particular linear and star polymers.

## 1. Introduction

Dendrimers are a relatively new class of macromolecules that can be prepared with extremely accurate control of architecture and molecular weight, thus being essentially monodisperse [1,2]. Dendrimers can be topologically described as being formed by concentric shells, or generations *g* growing from a central core, or branch point, with an *f* functionality (but a central unit with two functional groups are also found), therefore comprising *f* dendra of order *m*; here, *m* are the branches stemming from each trunk, while *p* bonds are present between the adjacent branch points, which have an *m* + 1 functionality.

Many applications in different fields are envisioned for these macromolecules, including drug delivery [3], catalysis, molecular electronics and nanomedicine [4], nucleic-acid transfection system [5] and theranostics [6]. However, their highly branched monodisperse structures with a well-defined and highly symmetrical geometry also provide stringent tests for analytical theories and molecular simulations to be compared with experimental data. In this review, we describe the theoretical and simulation investigations on the dynamics of dendrimers in dilute solution carried out with different methods. The observable quantities of interest discussed in the following are mainly mechanical properties, in particular the intrinsic viscosity [η], measured in the Newtonian regime at small shear rate $\dot{\gamma }$, (possibly together with the non-Newtonian behavior at high $\dot{\gamma }$), and the viscoelastic complex modulus measured under an oscillating shear stress, but we will also briefly discuss the dynamic structure factor measured in quasi-elastic scattering experiments.

Much emphasis will be placed on the intrinsic viscosity, since in dendrimers it has been found to show a maximum when plotted as a function of *g*. This behavior is present in poly(amidoamine), or PAMAM, dendrimers emanating from two functional groups instead of a single core (usually a diethylamino, or DEA, moiety) [1,2,7], in tri-polybenzylether, or Tri-PBzE, dendrimers [8] and in phosphorous-containing dendrimers having [OC_6_H_4_P(Ph)_2_ = N-P(S)] repeat units [9]. Moreover, a maximum of [η] was also found in non-regular hyperbranched PAMAM molecules at a somewhat larger molar mass than the PAMAM dendrimers [10]. On the other hand, no such maximum of [η] was found in nitrile- and amine-terminated poly(propyleneimine), or PPI, dendrimers with a diaminobutane (DAB) core having two functional groups [11,12]. Quite surprisingly, we note that to the best of our knowledge these are the only papers in the literature that systematically investigate the dependence of [η] on dendrimer generation, or equivalently on the polymer molar mass. Thus, it would be most useful to check whether the trend is also found for other dendrimers or hyperbranched polymers having different repeat units and different spacer lengths between adjacent branch points.

In the following, we briefly mention the basic features of the current analytical theories and simulations methods relevant to study the intramolecular dynamics of polymers, and then we review the results obtained by these methods. In the final section, we provide a summary and an outlook on the possible future theoretical and experimental work in this area.

## 2. Overview of Analytical Theories and Simulation Methods

### 2.1. Analytical Theories

The intramolecular dynamics of polymers are most often described through a set of stochastic Langevin equations for each bead in a coarse-grained model written in terms of their coordinates [13,14]. This equation expresses a balance between an inertia term and the forces acting on each polymer unit (atoms, monomers or beads depending on the adopted model). The latter forces account for the intramolecular elastic forces exerted by the other beads thanks to their connectivity, and may also take into account the exclude volume interactions, related with the bead covolume. In any case, this term couples the dynamic equations of each bead. In fact, the simplest polymer model that neglects these interactions, and which may somewhat apply to the unperturbed Θ state, corresponds to the so-called bead-and-spring chain, the conformation of which is equivalent to a random-walk model with the appropriate polymer topology; in this model, the elastic force acting on a given bead transmitted along the molecular backbone is simply proportional to its distance from the connected beads, just as in the Hookean elastic spring, while more complicated expressions apply in the presence of more sophisticated polymer models or in the presence of excluded volume interactions.

The stochastic dynamic equations also take into account the dissipative friction force with the solvent (the Rouse limit), but usually also account for the hydrodynamic interaction in the partial draining or in the impermeable coil limit (the Zimm limit) related to the drag effect of the solvent, which is assumed to be an incompressible fluid. For this reason, the motion of a given polymer strand affects the motion of other topologically distant strands through the induced motion of the solvent, which strongly affects the conformational relaxation of the polymer [13,14]. The hydrodynamic interaction is often modelled through the pre-averaged Oseen tensor for point-like friction beads, calculated through the average molecular conformation in terms of the averaged reciprocal distances $\left\langle r_{ij}^{-1}\right\rangle$ among the beads *i* and *j*. Moreover, these averages are usually obtained from the corresponding mean-square distances assuming a Gaussian distribution for the interbead distances, while they may be correctly calculated in computer simulations. A more sophisticated description of the hydrodynamic interaction accounts for the finite bead size, which may be particularly relevant at short interbead distances. In this case, the Rotne-Prager hydrodynamic tensor [13] can be used in simulation studies (see below). Note, however, that the latter, more accurate tensor would yield the same expression as the Oseen tensor in the pre-averaged approximation. Finally, the Langevin equation also accounts for the random Brownian forces; these forces have a zero mean, and a variance given by the Fluctuation-Dissipation theorem [14]. This term arises from the random collision with the solvent molecules, and accounts for the thermal bath at the chosen temperature, thus compensating for the dissipative effect of the friction.

While the inertia terms are neglected when dealing with the solution dynamics of molecules because of the very small values of the masses of atoms, of the repeat units or of the beads compared with their friction coefficients with the solvent (technically, one is dealing with an over-damped motion), in order to solve the dynamic equations, which are in any case coupled because of the intramolecular elastic forces even in the free-draining limit (Rouse limit), they must be decoupled through a transformation to normal coordinates that relax independently. This procedure is performed through the diagonalization of a non-symmetrical matrix having *N* × *N* dimension (*N* being the bead number), given by the matrix product of two different matrices: a symmetrical one accounting for the elastic forces, and a non-symmetrical one accounting for the hydrodynamic interactions. Such diagonalization, usually performed through standard numerical procedures, yields the relaxation times, which are proportional to the reciprocal of the eigenvalues, while the matrix collecting the eigenvectors gives the transformation of the bead coordinates into the statistically independent normal modes.

The normal modes relax independently with an exponential law, the characteristic times being given by the relaxation times. Each normal mode is also characterized by an integer mode number, indicated as *p* (*p* = 0, 1, 2, ... *N*), which makes it possible to order the eigenvalues in a decreasing order. The mode with *p* = 0 corresponds to the in-phase motion of the whole molecule, and thus to its diffusion, while the other indices describe the in-phase motion of the polymer strands comprising *N*/*p* beads in a half-wave motion. Therefore, an increasing *p* will describe increasingly shorter strands moving in phase, and thus with an increasingly short relaxation time. Incidentally, we note that this procedure also makes it possible to calculate the equilibrium averages of interest, in addition to the dynamical ones.

The dynamical quantities of interest include the intrinsic viscosity [η], given by

(1) $$\left[\eta \right]∼\frac{1}{N}\sum_{p=1}^{N}\tau_{p},$$

where τ*_p_* are the relaxation times, τ*_p_* ~ (2λ*_p_*)^−1^, with λ*_p_* being the above-mentioned eigenvalues or relaxation rates. We can also obtain the real and the imaginary part of the viscoelastic complex modulus [G′(ω)] and [G″(ω)], measured under an oscillatory shear deformation with the frequency ω

(2) $$\left[G^{\prime }\left(\omega \right)\right]∼\frac{1}{N}\sum_{p=1}^{N}\frac{{\left(\omega \tau_{p}\right)}^{2}}{1+{\left(\omega \tau_{p}\right)}^{2}};$$

(3) $$\left[G^{\prime \prime }\left(\omega \right)\right]∼\frac{1}{N}\sum_{p=1}^{N}\frac{\omega \tau_{p}}{1+{\left(\omega \tau_{p}\right)}^{2}},$$

where [G′(ω)] and [G″(ω)] are the storage and loss modulus, respectively, corresponding to the stored elastic energy and to the viscously dissipated energy within an oscillation cycle.

In linear chains, the relaxation times can be approximately expressed as τ*_p_* ~ (*N*/*p*)^3ν^, where ν is the Flory’s exponent, equal to ½ in the Θ state and ⅗ in a good solvent. By writing τ*_p_* = τ_1_/*p*^3ν^ where τ_1_ ~ *N*^3ν^ is the longest relaxation time, we obtain the Mark-Houwink-Sakurada Equation [13] from Equation (1):

(4) $$\left[\eta \right]=KM^{a},$$

where the molar mass is *M* ~ *N*, and *a* = (3ν)^−1^, so that *a* = 0.5 or 0.8 in the Θ state and in a good solvent, while the experimental values are 0.5 and ≅ 0.75, respectively. Additionally, the ω-dependence of [G′] and [G″] can be obtained as

[G′] ~ ω^2^, [G″] ~ ω for ωτ_1_ → 0; (5)

[G′] ~ ω^α1^, [G″] ~ ω^α2^ for 1 < ωτ_1_ < ωτ*_N_*, (6)

with α1 = α2 = (3ν)^−1^, the latter case corresponding to frequencies that probe the intramolecular dynamics.

Another dynamic quantity of interest is the Dynamic Structure Factor, which is best studied in quasi-elastic neutron scattering [15]. The dynamic structure factor *S*(*q*, *t*), where *q* = 4π sin(θ/2)/λ is the modulus of the scattering vector, θ being the scattering angle and λ being the radiation wavelength, is given by

(7) $$S\left(q,\;t\right)=N^{-2}\sum_{i}\sum_{j=1}^{N}\exp\left[-\frac{q^{2}}{6}\langle {\left|r_{j}\left(t\right)-r_{i}\left(0\right)\right|}^{2}\rangle \right]=\exp\left[-q^{2}Dt\right]\;\frac{1}{N^{2}}\sum_{i}\sum_{j=1}^{N}\exp\left[-\frac{q^{2}}{6}\langle r_{ij}^{2}\left(t\right)\rangle \right]$$

where *r_i_*(*t*) is the vector position of unit *i* at time *t*, while *D* is the diffusion coefficient of the center of mass and $\left\langle r_{ij}^{2}\left(t\right)\right\rangle$ is the mean-square distance of bead *j* at time *t* and of bead *i* at time 0 in a frame of reference diffused with the center of mass, which can be obtained from the eigenvectors and the relaxation times described before. The time decay of the *S*(*q*, *t*) is often characterized on the basis of the first cumulant Ω(*q*), given by the initial logarithmic slope

(8) $$\Omega (q)=-\frac{\partial }{\partial t}\ln{\left(\frac{S\left(q,\;t\right)}{S\left(q,\;t\right)}\right)|}_{t\to 0}$$

This quantity is also important, because, thanks to the *t*→0 limit, Ω(*q*) can be calculated either with or without the pre-averaging approximation to the hydrodynamic interaction, thus providing a way of assessing the error entailed in this procedure. The power-lay *q*-dependence of Ω(*q*) for linear chains can be summarized as follows:

(9) $$\Omega (q)=Dq^{2}\;\;\;\mathrm{for}\;qR_{g}\ll 1$$

(10) $$\Omega (q)=D_{\mathrm{bead}}q^{2}\;\;\;\mathrm{for}\;qR_{g}\gg 1$$

(11) $$\Omega (q)∼q^{3}\;\;\;\mathrm{for}\;qR_{g}\approx 1$$

where *D*_bead_ in Equation (10) is the diffusion coefficient of the single bead before it experiences the connectivity effects of the bonded beads, while Equation (11) is valid in the presence of the hydrodynamic interaction independent of the possible presence of the excluded volume.

### 2.2. Simulation Studies

Molecular simulations have been used for quite a number of years in polymer physics, as described in many excellent textbooks [16,17,18], and therefore, here we simply point out a few basic issues that will be relevant in the following.

The oldest simulation methods are the Monte Carlo methods, which aim to randomly sample the possible molecular conformations generated in the simulation in order to calculate the average relevant quantities of interest. New conformations can be generated by some random displacement of atoms (or beads) according to some definite rules that depend on the chosen model. The simplest one consists of placing the polymer on a regular lattice, often a simple lattice for a coarse-grained model, and then moving the units (or group of units) onto different lattice sites, provided they are empty so as to avoid overlaps or self-intersection of the molecule. This is computationally efficient, in that one simply has to keep trace of the occupancy or vacancy of each site. A different method adopts continuous space, and the random moves involve small local displacements of individual units, which is particularly efficient for dense systems such as dendrimers. In the latter case, the Metropolis algorithm is usually adopted: the energy change Δ*E* associated with the move due to bond stretching and non-bonded interactions, for instance, is calculated together with the corresponding Boltzmann weight ω = exp(−Δ*E*/*k*_B_*T*), then a random number ε uniformly distributed in the 0–1 range is selected, and finally the move is accepted if ε < ω. In this way, one can also accept moves that produce an energy increase with a finite probability. An important point is that in general, the chosen rules of motion should satisfy the detailed balance, i.e., a move must be chosen in an unbiased way, or more precisely, the reverse move must have the same probability as the direct one. This is not a necessity, but is a sufficient condition for obtaining system ergodicity, so that all accessible microstates in the relevant phase space (i.e., all microscopic conformations) are sampled with the same probability, thus avoiding any bias.

Another quite common simulation technique is the Molecular Dynamics method, whereby one is able to follow the time evolution of the system using the classical equations of motion (Newton’s equations). In this approach, each unit moves following the familiar equation *F* = *ma*, written as -∇25BD*V*=$m\ddot{R}$, where *V* is the potential energy of the selected model (accounting for bond-stretching, non-bonded interactions, etc.), *R* is the position of the unit, and *m* is its mass. This equation is solved numerically by assuming a discretized time with a very small time step with standard robust numerical methods. Since the basic equation is a second-order differential equation, two initial conditions are required, namely the starting trial coordinates and the starting velocities, randomly selected at the chosen temperature from the Maxwell-Boltzmann distribution. These simulations are usually carried out at a constant temperature, whose instantaneous value is determined by the average kinetic energy in a canonical ensemble. Appropriate algorithms are then used to keep the average temperature equal to the selected one (with fluctuations). The system is generally equilibrated in the initial part of the simulation trajectory, and then the quantities of interest are periodically sampled to calculate their statistical averages.

A variant of this method is the Brownian Dynamics method, which uses the stochastic Langevin equation previously mentioned, neglecting again the inertia term under the assumption of an over-damped motion. Note, incidentally, that in this case the Rotne-Prager tensor can be easily implemented, rather than the Oseen tensor, because pre-averaging is not needed. It should be noted that this method has a computational advantage compared to Molecular Dynamics in that it does not require the solvent to be explicitly modelled, since it is implicitly accounted for by the friction force and of the heat bath included in the random Brownian forces. The Langevin equation is then integrated numerically in time, and for an ergodic system, the relevant averages are obtained by a time average over the simulated trajectory.

## 3. Theoretical and Simulation Results for Directly Observable Quantities

### 3.1. Relaxation Times and Intrinsic Viscosity

#### 3.1.1. Analytical Approaches

**Figure 1:**
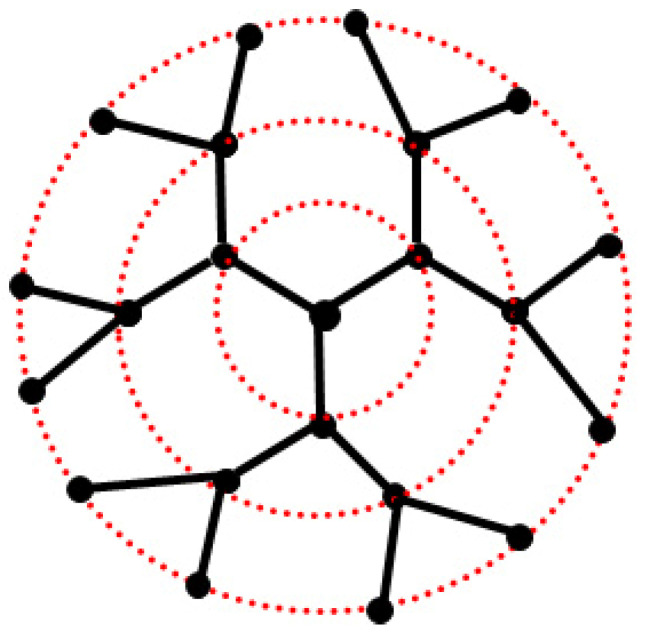
A schematic drawing of the topology of a dendrimer. In this coarse-grained model, there is a single tri-functional core (*f* = 3), binary dendra (*m* = 2) and a single bond connecting adjacent branch points (*p* = 1), while the dotted circles indicate the successive generations *g*. This is a second-generation dendrimer (*g* = 2) if *g* is numbered from 0 onward, but in some cases, if the numeration starts from 1, it will be denoted as a third-generation dendrimer.

The first investigation of the intramolecular dynamics of dendrimers was carried out by La Ferla [19] within the framework of the Rouse-Zimm approach. The modelled dendrimers had a ternary core (*f* = 3) and binary dendra (*m* = 2), as shown in Figure 1, resulting in a variable number of bonds *p* between consecutive branch points, while the generations *g* were numbered from 0 onward. The intramolecular dynamics were described on the basis of the Langevin equation. As for the chosen conformational model, which determines the intramolecular elastic forces acting on each bead, La Ferla adopted a freely rotating model, whereby the connected springs associated with each chain segment and which have one bead in common exhibit fixed bond angles that may be different depending on whether the bead is the central core, another branching point, or is part of a linear portion; the simplest freely jointed model was recovered by setting the average cosines of all the bond angles to 0. Only topologically short-ranged interactions were included, while long-range excluded volume interactions were neglected. The scalar products between the bond vectors determine the intramolecular elastic forces through an incidence (or connectivity) matrix, depending only on the molecular topology. From a conformational viewpoint, La Ferla was able to obtain analytical results for the radius of gyration $R_{g}$ in the freely jointed dendrimer that were equivalent to results obtainable from a random walk model, as a function of *f*, *m*, *g* and the number of bonds *p* between adjacent branch points [19]. Here, we are only interested in the dynamical properties, starting from the spectrum of relaxation times and the intrinsic viscosity [η]. The relaxation spectrum shows a large degeneracy in the relaxation times τ*_p_*, which become a universal function at high *g* if they are plotted as a function of *p*/*N* (see Section 2.1). Considering dendrimers having *p* = 1, La Ferla was also able to determine the degeneracy of the collective modes as a function of *f*, *m*, *g* for dendrimers, that is, based only on the dendrimer topology. The intrinsic viscosity [η] can thus be obtained through Equation (1), as it is mainly controlled by the longest relaxation times of the collective modes. Unfortunately, La Ferla did not report the *g*-dependence of [η] for a comparison with experimental data, but since [η] is proportional to $R_{\eta }^{3}$/*M* based on the known coefficients, where *R*_η_ is the viscosimetric radius and *M* is the molar mass, he showed that the calculated *R*_η_ did not follow a power-law dependence on *M*, unlike what is found for linear chains and star polymers. Moreover, the *R*_η_ values, calculated for dendrimers with *f* = 3 and *m* = 2, exceeded the experimental values, with higher values corresponding to higher generation *g*. The reason for this discrepancy was tentatively attributed to a small incomplete branching at high *g* of the experimental samples, which was also implied by the small but non-negligible polydispersity, which would affect the viscosimetric radius much more that the radius of gyration.

The spectrum of relaxation times and the intrinsic viscosity were later investigated while also considering the effect of good solvent expansion [20] in view of the large covolume effects that are present in the sterically crowded dendrimers. Intramolecular expansion was calculated through self-consistent minimization of the intramolecular free energy, accounting for the configurational entropy loss experienced by the swollen molecule and for the covolume two-body interactions. In turn, the latter interactions are calculated on the basis of the pairwise contact probability among the beads, assuming a Gaussian distribution of the (perturbed) inter-bead distances. Both these free-energy contributions can be written in terms of the scalar products among all the bond vectors connecting the beads for the topology of interest [20,21,22]. As a result, the equilibrium state is determined, which corresponds to the optimal compromise between the repulsive covolume interactions, which tend to non-affinely swell the molecule, and the elastic penalty opposing it as a result of the entropic configurational loss. This approach led to an asymptotically finite expansion dictated by the finite, and quite small, span of each dendron. Moreover, the optimized scalar products among the bond vectors also yield the elastic forces acting on the beads and the inter-bead distances required to account for the hydrodynamic interaction. However, while the agreement with the experimental values for the PPI dendrimers were quite satisfactory for $R_{g}$, the viscosimetric radius obtained from the calculated [η] was found to exceed the experimental data at high values of *g*, which is in keeping with the La Ferla’s results. Moreover, the intrinsic viscosity [η] did not show any maximum if plotted as a function of *g*, and only the phantom molecule (which corresponds to a random walk) hinted at the possible presence of such a maximum, and only for unrealistically high *g* values. In the same paper [20], it was also shown, however, that slightly attractive pairwise interactions among the beads, resisted only by configurational entropy, could indeed lead to a maximum in [η]. Interestingly, such weak attractive interactions, corresponding to a slightly negative binary cluster that is integral among the beads [13], are actually required in order to achieve an unperturbed Θ state, whereby the temperature produces a vanishing second virial coefficient between the molecules that is measured, for instance, in osmometry and in light scattering experiments [23]. Because of the molecular topology, in branched systems, the interactions between two molecules also entail repulsive three-body interactions among the beads; in fact, two beads of different molecules cannot freely approach one another because of the covolume of a topologically neighboring third bead. Such intermolecular repulsion must be compensated by a slightly negative binary cluster that is integral to achieving the Θ state [23]. Therefore, it can be concluded that the maximum [η] obtained in Reference [20] corresponds to what was calculated for the Θ state. One general issue related to this point is that, in overcrowded systems such as dendrimers at high values of *g*, the excluded volume effects are effectively screened out by the locally dense environment, similar to what is found in polymer melts. As for the spectrum of relaxation times, this has been analyzed in more detail in a subsequent paper, discussed below in connection with the calculations of other dynamical observables [24], where the degeneracy of the relaxation times, related to the topological symmetry of the molecules alone was fully determined as a function of *g*, *f*, *m* and *p*.

The analysis of the normal modes of motion of dendrimers and the multiplicity of relaxation times related to molecular symmetry was investigated independently by Cai and Chen [25] with respect to the Rouse free-draining limit, that is, neglecting hydrodynamic interactions. Moreover, the intramolecular excluded-volume interactions were ignored, so that the molecule could be conformationally described as an appropriate random walk. In this way, many quantities of interest can be analytically calculated, such as $R_{g}$, for instance [19]. More interestingly, in this way, the dynamical problem can be solved quasi-analytically, and the normal modes of motion can be obtained explicitly together with the relaxation times (or at least the collective ones) for the low-generation dendrimers, and then, by extension, for higher *g* values. The degeneracy of the relaxation times was largely determined in this way (see also Reference [24] for a more systematic list), while the intrinsic viscosity could also be analytically determined as a function of the generation, *g*, in consideration of dendrimers with a tri-functional core (*f* = 3) and binary dendra (*m* = 2); in keeping with the other investigations mentioned above, [η] was found to increase monotonically with *g*, with no indication of any maximum [25]. In a subsequent paper [26], Cai and Chen investigated the intrinsic viscosity [η] of dendrimers in more detail, adopting the variational approach of Fixman [27] to tackle this problem, the exact solution of which is a formidable task, usually requiring some approximation such as the pre-averaged approximation. Since the effect of this approximation on the calculation of [η] is unknown, but can be large in dense systems, Fixman proposed two different approximations that could bracket the “true” values, rather than the pre-averaged one, which provides an overestimation of [η]. Cai and Chen adopted the Rotne-Prager tensor to model hydrodynamic interaction, since it is more accurate that the Oseen tensor, but this introduced a further parameter—hydrodynamic bead diameter. If this value is equal to the excluded-volume bead diameter used to account for the bead covolume, no maximum can be obtained for [η] for either the upper or the lower limit. If, however, the hydrodynamic bead diameter is close to the segment length, while the covolume bead diameter is very small (thus approaching the Θ state), a shallow maximum can indeed be obtained. It can be noted that this procedure is quite ad hoc, and no clear molecular basis for this difference can be given on physical grounds. This problem was investigated again by the same group with Monte Carlo simulations [28], as described in the next section.

The theoretical study of dendrimer dynamics was subsequently carried out by Biswas’ group employing the standard Rouse-Zimm approach with hydrodynamic interaction and using the pre-averaged Oseen tensor [29]. Biswas et al. adopted a semiflexible conformational model by imposing restrictions on the direction and orientations of consecutive bond vectors, somehow accounting locally for the excluded-volume effects. The conformational model yields equilibrium quantities such as $R_{g}$, for instance, and determines the elastic force terms in the Langevin equation. The restrictions on the direction and orientation of the bond vectors are applied by fixing the bond angle values formed by the vectors stemming from a branch point in polar coordinates using appropriate spherical harmonics, thus describing the local correlation between consecutive bonds of different generations (since *p* = 1, see Figure 1) and allowing for more compressed or more expanded conformations. The elastic forces are then evaluated based on the average scalar products between the bond vectors, so that finally the spectrum of the relaxation times and of the normal modes of motion can be obtained. While the multiplicity of the relaxation times is not affected, the first result is that the rigidity constraints of the semiflexible dendrimers produces longer relaxation times for the local modes, but does not greatly affect the relaxation times of the collective modes. Interestingly, the resulting intrinsic viscosity shows a well-defined maximum when plotted as a function of *g*, this maximum being present at *g* = 7 quite independently of stiffness (generations are numbered from *g* = 1 onward), and being only slightly shifted to *g* = 6–7 with more compressed conformations. It should be pointed out that such *g* values (calculations were carried out up to *g* = 10) are quite high, producing a very high intramolecular density that is only achieved for PAMAM dendrimers that have a relatively long and flexible spacer between adjacent branch points and an EDA central unit with two functional groups, such that a value of *g* = 10 was realized. It is noteworthy that a pronounced maximum was also obtained in the same paper [29] for the model adopted by La Ferla [19], carrying out calculation for up to *g* = 10 (La Ferla considered the *g* = 6 case, at most); furthermore, in this case, a maximum of [η] was obtained for *g* = 7. It may therefore be concluded that in other cases, too (for instance in References [20,25,28]), such a maximum could indeed be found at *g* values higher than those considered. Therefore, the existence of a maximum of [η] as a function of *g* could be a general feature of current approaches that are close to the ideal Θ state, even though the quantitative agreement would often be quite poor, at least at this location.

Biswas’ group also investigated the dynamical properties of randomly hyperbranched polymers along the same lines [30,31]. The hyperbranched polymers were closely similar to dendrimers, but were built using a growth algorithm starting from a three-functional core (*f* = 3) and binary branches (*m* = 2) after each bond. However, at each generation, one end unit was randomly selected as a dead end, wherefrom no further branching (or linear bonding) took place [30], while a limited flexibility was introduced in the same way as previously described in References [29,32]. In the second paper on hyperbranched polymers, the elastic forces were accounted for through the elastic springs associated with each bond and through a local excluded volume term between beads belonging to the same or to the adjacent shell only with two different parameters [31]. It may be pointed out again that this description would not account for the long-range excluded volume effects of linear polymers, since it would simply renormalize the segment length of an equivalent random walk chain. However, it could be used for dendrimers, assuming that the covolume effects are effectively screened out in dense systems. The dynamical equations, which account for hydrodynamic interactions through the pre-averaged Oseen tensor, yield the spectrum of relaxation times and the normal modes of motion for different values of the excluded volume parameters. The intrinsic viscosity [η] is then obtained as a function of *g*; a clear maximum is seen for *g* = 5 in the case of regular dendrimers with a significant excluded volume interaction, while in the analogous randomly hyperbranched polymer the maximum takes place for *g* = 6 and is less pronounced with a weaker decrease of [η] at higher generations. Moreover, in the hyperbranched polymers, the magnitude of [η] increases with decreasing strength of the excluded volume interaction, ultimately approaching the values of star polymers with the same number of beads, with a monotonous increase of [η] at an increasing molar mass.

**Figure 2:**
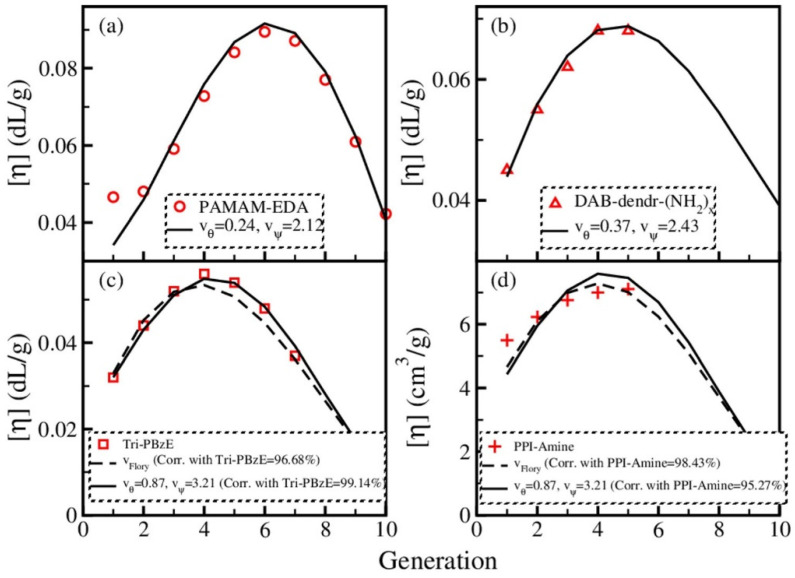
Plots of the experimental and calculated intrinsic viscosity [η] for different dendrimers as a function of generation. The experimental results are for PAMAM dendrimers with an EDA core (**a**) [1], for an amino-terminated PPI dendrimer with a DAB core, indicated as a DAB-dendr-(NH_2_) (**b**) [11], for a Tri-PBzE dendrimer (**c**) [8] and for a PPI dendrimer with a DAB core (**d**) [12]. In the panels, ν_θ_ and ν_ψ_ are the two excluded-volume parameters for beads belonging to the same or to the adjacent generation (see the text and Reference [33] for more details), while ν_Flory_ is the parameter estimated from Flory’s mean-field approximation [33]). Reprinted with permission from Rai, G.J.; Kumar, A.; Biswas, P. Dynamics of dendrimers with excluded volume: a comparison with experiments and simulations. *J. Rheol.* **2016**, *60*, 111–120, doi:10.1122/1.4937378. Copyright 2016, The Society of Rheology.

Dendrimer dynamics were also discussed in a further paper by Biswas’ group [33], again using a pre-averaged hydrodynamic interaction and accounting for the excluded volume between the nearest non-bonded beads only. The strength of this interaction was characterized by a parameter derived from Flory’s mean-field approximation through minimization of the molecular free energy accounting for the configurational entropy and an excluded volume term estimated in the mean-field approximation through the volume pervaded by the polymer (a sphere having a radius equal to $R_{g}$). Because of that, the excluded-volume parameter depends on the number of beads, on the number of the nearest non-bonded interactions and on the sum of the distances among the bead pairs, hence on the dendrimer generation. An alternative approach adopted in the same paper consists of adopting the geometrical procedure proposed by the same group [31] in terms of two parameters for beads belonging to the same or to the adjacent generation only (see before). With either method, in order to account for the excluded-volume parameter, a maximum of [η] is obtained for relatively small *g* values. In fact, using the geometrical procedure with a small excluded volume parameter, the maximum can be seen at *g* = 6, with a small shift at higher *g* for a weaker excluded value strength; when using the parameter obtained with Flory’s mean-field approximation, it takes place at *g* = 4 (the generations are numbered from *g* = 1 onward). By tuning the excluded-volume parameters of the geometrical approach, Biswas et al. [33] were also able to satisfactorily reproduce a large number of experimental and simulation results on the intrinsic viscosity of dendrimers. Figure 2 summarizes the experimental results mentioned in the introduction and the fitting results of Biswas et al. [33].

As a final theoretical approach, we should also mention an entirely different method for calculating the intrinsic viscosity of dendrimers based on a sort of mesoscopic molecular description, rather than a fully microscopic one as used before. The theory was first proposed in terms of a simple two-zone model of dendrimers [34]. In this model, the dendrimer is described as being formed of a dense core impermeable to the flow field, where the solvent molecules are effectively trapped (thus adopting the Einstein result for the viscosity of a suspension of spheres [13]), and a thin outer region where the solvent drains freely (i.e., in the Rouse limit). Assuming spherical symmetry and a Gaussian radial density profile, the dependence of [η] on *g* could be calculated in quantitative agreement with the experimental results for PBzE dendrimers, in particular with respect to the presence of the maximum and its position. The theory and the general mesoscopic molecular picture of polymers was subsequently developed in greater detail [35], introducing a drag function in order to describe the local volume fraction of the solvent flowing with the polymer, and a drainage function related to the solvent that moves through the polymer. Both functions are calculated from a spherical symmetric density profile, which is now determined on the basis of appropriate Monte Carlo simulations for the topologies of interest using the Lennard-Jones potential for the non-bonded interactions. The results of these calculations favorably compare semi-quantitatively with experimental data from polymers of different topologies (linear, ring, star polymers) and for the dendrimers (PAMAM, PBzE, PPI) with only a few adjustable parameters. We note that the connection of this model with a more microscopic one is quite unclear, and would require a more detailed investigation, as also indicated at the end of this review.

#### 3.1.2. Simulation Methods

The first simulation study investigating the intrinsic viscosity of dendrimers and of hyperbranched polymers was probably carried out in 1998 using Metropolis Monte Carlo simulations [36]. The polymers were built starting from a trifunctional B_3_ core by sequentially adding AB_2_ monomers assuming definite reaction probabilities at the ends of each unit. By tuning these a priori probabilities, both dendrimers and hyperbranched polymers with some dead ends could be obtained. The chosen monomers corresponded to a branched alkane, described on the basis of the Rotational Isomeric Scheme (RIS) [37] for the rotations around single bonds, but they effectively adopted a phantom-chain model (bond crossing was not forbidden). The excluded-volume interactions were neglected, as well as the hydrodynamic interactions. The intrinsic viscosity was then calculated through the Flory-Fox equation [η] ~ $R_{g}^{3}$/*N*, with $R_{g}$ being the radius of gyration obtained by the simulations and *N* being the total number of monomers. Quite surprisingly, in view of the above-mentioned approximations and the inadequacy of using the Flory-Fox equation noted in other studies [34,38], this approach yielded a maximum of [η] for *g* = 3 for regular dendrimers (*g* was numbered from 0 onward), in a range consistent with the experimental data. A similar maximum was also predicted for hyperbranched molecules, with the maximum progressively shifting to a somewhat larger molecular weight corresponding to g ≈ 4–5 of the respective dendrimers with a decreasing branching pattern, and with larger [η] values than the corresponding dendrimers [36].

The calculation of the spectrum of relaxation times and of intrinsic viscosity was later investigated in more detail by Cai and Chen [28], with an analysis of the approximations involved in the analytical studies. The dendrimer conformation was modelled by standard Monte Carlo simulations in continuous space for a freely jointed model with *g* = 3 and *m* = 2 (see Figure 1) and with a single bond between adjacent branch points in order to calculate averages of interest, including those yielding the elastic potential and the elements of the hydrodynamic tensor, both in the simple Oseen form and in the more sophisticated Rotne-Prager form. The dynamical equations were then solved numerically, producing the spectrum of relaxation times and the normal modes of motion. The relaxation times showed the expected degeneracies independently of the strength of the excluded volume interaction. As for the intrinsic viscosity, the pre-averaged Oseen tensor led to values that somewhat exceeded both the upper and the lower bounds calculated using Fixman’s variational method with the Rotne-Prager tensor [27]. In any case, no maximum of [η] was obtained as a function of *g* using the same bead diameter for the hydrodynamic interaction and for the excluded volume [28].

A more complete simulation study of dendrimer dynamics was later performed by Mansfield in a seminal paper [39], adopting lattice Monte Carlo simulations for the dendrimers. The issue of the simulation of the transport properties, including the intrinsic viscosity in particular, was dealt with using an analogy with electrostatic and random-walk statistics, so that the Navier-Stokes equation was transformed into the Laplace equation, while the intrinsic viscosity was proportional to the trace of the polarizability tensor, even though the proportionality constant depends on the shape of the body. It is not clear, however, how much this analogy depends on the step length of the random walk, and on the assumption of a continuous solvent medium, as adopted in the Rouse-Zimm approach. The simulations were carried out for dendrimers on a diamond lattice, assuming that seven bonds were present between adjacent branch points under excluded volume conditions, and showed a well-defined maximum of the intrinsic viscosity for *g* = 6 [39]. Unfortunately, the applied methodology violated the detailed balance [40], potentially casting some doubt on the calculated properties. Further simulations by Mansfield and Jeong [41] took this issue into account and overcame the original problem by using a different criterion for accepting the trial moves. The result was that the former Mansfield’s results were significantly affected by the detailed-balance violation for phantom-chain dendrimers, but were qualitatively valid provided the exclude volume interactions were accounted for, so that in this case a sharp maximum of [η] was calculated for *g* = 6–7. No direct comparison with the experimental data was attempted, however, as the main emphasis was on the conformational equilibrium properties.

Another simulation study of the dynamical properties of dendrimers was carried out by Lyulin et al. using Brownian Dynamics simulations of dendrimers under a shear flow [42]. Again adopting a dendrimer model with a trifunctional core (*f* = 3) and binary dendra (*m* = 2) comprising either one or two bonds between adjacent branch points (*p* = 1 or 2), the equations of motion accounted for the hydrodynamic interaction with the Rotne-Prager tensor. The interaction potential among the beads was modelled on the basis of Lennard-Jones potential, with parameters that reproduced the unperturbed Θ state in linear chains, while a rigid constraint was applied to the bond lengths, and the solvent velocity in one direction was given in terms of an applied shear rate $\dot{\gamma }$. The intrinsic viscosity [η], calculated from its definition as the ratio between the appropriate non-diagonal component of the stress tensor and the applied shear rate in the limit $\dot{\gamma }$→0, displayed a shallow maximum for *g* = 4–5. At a significantly higher shear rate, a shear thinning behavior was obtained, such that [η] was found to decrease with an increase of $\dot{\gamma }$ according to the power-law $\dot{\gamma }$^−1/3^, with a concomitant significant increase in the molecular size in the shear direction, as expected. Hyperbranched polymers were also investigated using the same Brownian Dynamics simulations [43], with the polymers being built by using the same algorithm as in Reference [36]. A maximum of the intrinsic viscosity was again observed for dendrimers and for hyperbranched polymers with a large branching degree, while the shear thinning behavior was again detected at a large shear rate, although the simulations did not allow to detect a clear power-law dependence of [η] from $\dot{\gamma }$.

The intrinsic viscosity of dendrimers has also been modelled using Molecular Dynamics methods [44], adopting a coarse-grained model of dendrimers in explicit solvent, modelled as Lennard-Jones particles. Dendrimers were described up to *g* = 7 with a trifunctional core (*f* = 3), and binary dendra (*m* = 2), comprising a single harmonic spring between consecutive branch points (*p* = 1), while the non-bonded beads interacted through Lennard-Jones potential. The shear viscosity was calculated with the Green-Kubo formula as the integral over time of the stress autocorrelation function, using the off-diagonal terms of the stress tensor, which in turn were obtained from the momenta of the particles, and on the interparticle forces and distances. The intrinsic viscosity thus calculated, which is in principle quite computationally demanding, showed a clear maximum at *g* = 5, and in general compared very well with the Brownian Dynamics simulations [42].

A different approach was later subsequently by Freire et al. [45], who used coarse-grained Monte Carlo simulations in continuous space for dendrimers with a single core or a central unit with two functional groups and tri-functional branch points, comprising a further bead between adjacent branch points of the dendra so as to avoid bond crossing. Furthermore, the beads interacted through a hard-sphere potential with an appropriate bead diameter, which was indirectly related to the solvent quality. The starting conformations for the simulations were obtained through a lengthy procedure involving a preliminary atomistic Molecular Dynamics simulation using a standard force-field (the CVFF force field) for a few specific dendrimers [45]. A careful ad hoc strategy was used in these runs carried out in vacuo, but eventually the modelled $R_{g}$ reproduced the experimental value. In this way, a reasonable set of conformations was achieved, wherefrom a systematic coarse-grained sampling of relevant atoms (in particular the branch points) provided the starting coordinates of the beads for the Metropolis Monte Carlo runs. These simulations, which were much more efficient than the atomistic Molecular Dynamics runs for sampling the phase space of the system, were performed by small random displacements of a randomly selected bead, provided the resulting bond lengths were consistent with the distribution inferred from the atomistic Molecular Dynamics runs. These simulations yielded the conformational averages (for instance, those involved in the pre-averaged hydrodynamic interaction) required to calculate the intrinsic viscosity according to the lower bound of the variational procedure introduced by Fixman [27], assuming that a frictional radius of each bead had been appropriately chosen. The intrinsic viscosity plotted as a function of *g* did indeed show a maximum, which was quantitatively consistent with the experimental data [45]. It should be noted, however, that the ratio between the bead friction radius and the bead hard-sphere radius required for dendrimers appears to be much larger than that required in linear chains, which suggests a different non-draining behavior. This point would require a more detailed investigation, in our opinion; in fact, with these dense systems, the assumption of the solvent treated as a continuum that pervades the inner part of the molecule may be incorrect, while a different treatment of a few discrete molecules would be required, with a physically different approach.

Subsequently, Freire et al. improved their model somewhat by applying a few corrections of varying importance [46]. The first correction, and probably the most important one, was the addition of the contribution of an individual friction bead, which could be important for small molecules. This term amounted to assuming that the friction forces are distributed on the bead surface, rather than acting on the bead centers. The second correction amounted to incorporating a realistic distribution of the internal angle formed by the bonds connecting adjacent branch points. The third correction involved adopting a more realistic distribution of the distances between the adjacent branch points in order to better account for the presence of the solvent molecules. The simulation results again provided the observed maxima of [η] as a function of *g*, and were generally in very good agreement with the experimental data. Moreover, the optimized bead friction radius was generally more reasonable than what was required by Reference [45], even though its value, which was larger than that of the hard-sphere radius, was still not fully clear. Subsequently, these parameters were further revised [47], but the relative size of these two radii was not qualitatively affected. It should also be noted that in a subsequent paper, Freire et al. [48] also investigated the effect of pre-averaging on the calculated intrinsic viscosity obtained adopting the same model as in Reference [46]. The pre-averaged approximation turned out to be inaccurate, especially at high *g*, producing results that were too large by a factor greater than 2 at the highest values of *g* (*g* = 7).

In a later simulation study [49], Brownian Dynamics simulations were used to investigate the dynamics of dendrimers adopting a finite-extensible nonlinear elastic (i.e., non-Hookean) FENE potential for the springs connecting the adjacent branch points. Excluded-volume interactions were ignored, while the hydrodynamic interaction was modelled through the Rotne-Prager tensor. Each spring was assumed to stand for a given number of statistical segments (or better, Kuhn steps), which were entered as a parameter in the expression of the FENE potential, so that different spacers between the branch points could be effectively modelled. The viscosity was then calculated as the ratio between the applied stress tensor and the shear rate $\dot{\gamma }$, so that [η] was obtained within the limit $\dot{\gamma }$→0. The intrinsic viscosity plotted vs. the generation was found to show a maximum at *g* = 3, which is a bit less than, although still close to, what was observed experimentally. It is also interesting to point out that a significant effect of shear rate on viscosity was detected upon increasing $\dot{\gamma }$. In particular, the intrinsic viscosity first exhibited a minor decrease when ${\dot{\gamma }\tau }_{\eta }$ ≈ 1 (here, $\tau_{\eta }$ is a characteristic relaxation time derived from the zero-shear intrinsic viscosity), then a minor increase with a small shear thickening effect within a decade in the applied frequency, and finally a pronounced shear thinning effect at lager $\dot{\gamma }$ with a power-law scaling behavior so that [η] ~ $\dot{\gamma }$^−α^ with α ≈ 0.55–0.6. Interestingly, the same pattern of shear thinning, followed by shear thickening and then by a much larger shear thinning was previously predicted for heavily branched star polymers with long arm lengths using a different approach [50]. In the latter study, an analytical approach made it possible to describe the behavior of star polymers under shear, considering both the excluded-volume and the pre-averaged hydrodynamic interaction through the stochastic Langevin equation under the constraint of a constant contour length, somehow consistent with, but different in detail from, the approach of Reference [49]. These changes of the intrinsic viscosity with increasing applied $\dot{\gamma }$, starting roughly at the same reduced shear rate ${\dot{\gamma }\tau }_{1}^{0}$ ≈ 1, where $\tau_{1}^{0}$ is the longest relaxation time of the chain under a very small $\dot{\gamma }$ (→0), were attributed to the incipient arm unwinding, followed by a change in the draining regime due to the arm deformation, and eventually to a decrease of the hydrodynamic interaction due to the full arm deformation of the arms in the flow direction with an asymptotic power-law $\dot{\gamma }$^−α^ dependence with α = 2/3 [50]. Such physical effects are also at play in the case of dendrimers, as also briefly noted in Reference [49].

### 3.2. The Viscoelastic Complex Modulus and the Dynamic Structure Factor

#### 3.2.1. Analytical Approaches

**Figure 3:**
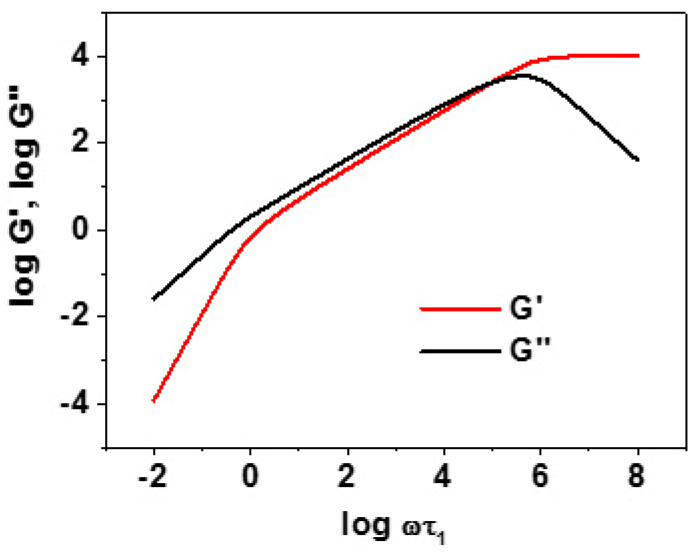
Plot of the real part G′ and the imaginary part G″ of the viscoelastic complex modulus for a linear freely jointed chain with 10^4^ repeat units. Here, the low-frequency behavior of Equation (5) can be clearly seen for ωτ_1_ < 1, while at higher ω, the power-law behavior of Equation (6) can clearly be seen. Finally, at very high ωτ_1_ (>10^6^ for this linear chain), the results are not physically realistic, since G′ and G″ would probe the intramolecular dynamics at length and time scales smaller that of a single spring.

In his seminal paper on the intramolecular dynamics of dendrimers, La Ferla also calculated the viscoelastic complex modulus for dendrimers modelled as described previously [19]. In order to better understand the discussion in this section, we report in Figure 3 the calculated results for a linear freely jointed chain in the partial draining regime (Zimm limit).

At a very low frequency, such that ωτ_1_ << 1, with τ_1_ being the longest relaxation time, the power-law relationships of Equation (5) clearly applied, with G′ being much smaller than G″, showing a predominantly viscous response with a negligible elastic one, as also shown by linear chains in Figure 3. On the other hand, no power law was found in the log–log plot of G′ and G″ vs. ω at higher frequencies, unlike what was predicted by Equation (6) and observed in experimentally linear and star polymers (see again Figure 3). Thus, La Ferla found a continuous and smooth change of slope of the curves related to the extensive dendrimer branching, or, more precisely, to the large degeneracies of the relaxation times discussed in Section 3.1.1. The main reason for this behavior is the small span of the individual dendra, and in particular the short path from the core to the terminal beads (seven steps at most for the largest generation, *g* = 6, if the starting generation is labelled *g* = 0), which is consistent with what has already been pointed out by one of us for star polymers [51]. Moreover, the calculated dynamic structure factor displayed a simple exponential decay as a function of *t* for $qR_{g}\ll 1$; that is, for distances of observation much larger than the molecular size, due to the diffusion of the center of mass. However, deviations from simple exponential decay were observed for larger q values, probing the intramolecular dynamics. The first cumulant was analyzed by plotting $\Omega \left(q\right)/q^{3}$ (see Equation (11) as a function of $qR_{g}$ for $qR_{g}$ of the order of 1 to detect deviation from the constant value shown by linear polymers. Indeed, a minimum of the exact $\Omega \left(q\right)/q^{3}$ (i.e., calculated without the pre-averaging approximation) was found at $qR_{g}$ ≈ 2, which is clearly related to the branched molecular structure, as it was also found in star polymers at the same position, albeit with a much shallower depth. Thus, the minimum became increasingly deep with increasing generation number, while the deviations from the pre-averaged values (whose minimum occurs at $qR_{g}$ ≈ 2.5) increase somewhat; it may thus be concluded that, as expected, pre-averaging leads to larger errors with higher degrees of branching. Such theoretical predictions are in semi-quantitative agreement with neutron spin echo experimental results obtained for dendrimers with a six-functional core (*f* = 6) and binary dendra (*m* = 2) with relatively long and flexible spacers between adjacent branch points [52]. In fact, a deep minimum was measured in the plot of $\Omega \left(q\right)/q^{3}$ as a funtion of $qR_{g}$. The minimum occurred at $qR_{g}$ ≈ 2.5–3, and thus was close to the calculated value, although somewhat deeper, while the increase of $\Omega \left(q\right)/q^{3}$ at $qR_{g}$ smaller than 2.5 was sharper than calculated, but smoother at higher $qR_{g}$ values. We note, incidentally, that in the same paper [52], the corresponding linear polymer followed the theoretical prediction of Equation (11). Furthermore, it should be added that in a previous paper of the same group [53], analogous neutron scattering experiments carried out on PAMAM dendrimers (which had a much shorter spacer length than the sample mentioned previously) with generations up to *g* = 8 only displayed a diffusive behavior, while the intramolecular dynamics were too slow to be effectively recognized.

A closely related theoretical approach was adopted by Blumen et al., who modelled Gaussian Generalized Structures (GGS) of beads connected by harmonic springs [54], in particular describing polymers in the Θ state and focusing on polymer deformation under an external field [55,56,57]. A first study modelled dendrimers using the stochastic Langevin equation, neglecting the hydrodynamic interaction (free draining regime) [55]. In this case, too, no power-law behavior of [G′] and of [G″] was found as a function of the applied frequency ω in the intermediate ω range, even though some hints of such functional dependence could be tentatively glimpsed for *g* = 9 (generations were numbered from *g* = 1 onward). The introduction of the pre-averaged hydrodynamic interaction into the GGS model was subsequently described [56], while the corresponding effect on the viscoelastic complex modulus was subsequently considered [57]. In the latter paper, the intramolecular dynamics, investigated at intermediate ω values, as noted previously, did not show any clear power-law dependence of [G′] and of [G″] on the applied frequency ω, but rather a near logarithmic one [57]. Introduction of a local stiffness in this model [58,59] in a way quite similar to that adopted by La Ferla [19], but in the free-draining regime, did not significantly change the qualitative picture, and again no power-law dependence of [G′] and of [G″] on ω was detected, which is in keeping with the La Ferla’s results. Even in these cases, we note that the small span of the dendra modelled as phantom chains, close to the Θ state, does not make it possible for the power-law dependence to actually be displayed.

Another investigation into the intramolecular dynamics of dendrimers and randomly hyperbranched polymers was carried out in Reference [60] in consideration of elastic springs for the bonds with restrictions on the bond angles, thus corresponding to a freely rotating model, and neglecting excluded volume interactions. Accordingly, the conformational model and the dynamic equations were the same as those adopted by La Ferla [19]. In this case, too, the dendrimers had a trifunctional core (*f* = 3) and binary dendra (*m* = 2), with a single bond between adjacent branch points (*p* = 1), while the hyperbranched polymers were built stepwise by randomly selecting a terminal vertex at each step and adding two branches to it, thus generating two further terminal vertices. The calculations for the randomly hyperbranched molecules were then averaged over a very larger number (10^4^–10^5^) of random realizations. The viscoelastic complex modulus calculated by this model did not show any clear power-law dependence in the intermediate ω range, which is consistent with the results previously obtained by La Ferla [19], which is hardly surprising, since the same conformational model was adopted. More interestingly, the randomly hyperbranched molecules only showed very minor, if any, differences compared to regular dendrimers when the comparison was carried out with the same number of beads or molecular mass. Accordingly, the presence of defects in the dendrimer structure does not lead to any significant differences in intramolecular dynamics.

A subsequent study of the viscoelastic complex modulus was later carried out by our group [24] by exploiting the above-mentioned equilibrium results [20] when investigating the conformational behavior and intrinsic viscosity of dendrimers in a good solvent. While the low-frequency power-law behavior of Equation (5) was again observed, in the presence of good solvent expansion, the increased breadth of the spectrum of relaxation times for high-generation dendrimers again yielded a power-law in the intermediate frequency range 1 < ωτ_1_ < ωτ*_N_*, with [G′] ~ ω^α1^ and [G″] ~ ω^α2^ (see Equation (6) and Figure 3 for a comparison). However, this power-law could not be clearly detected in dendrimers comprising a single bond between adjacent branch points (*p* = 1), and only in those with *p* = 2; in other words, a power law is only apparent in this frequency range provided the dendron span is large enough, or better, provided the core-to-end bead distance is great enough. In this case, the above-mentioned α exponents had the values α1 = 0.55 and α2 = 0.46, as compared with the predicted asymptotic values α1 = α2 = (3ν)^−1^ as noted after Equation (6), yielding a common exponent equal to 23 = 0.67 in the Θ state and 59 = 0.56 in a good solvent (but in finite chains α1 ≳ α2 [51]). In the same paper [24], the dynamic structure factor was also calculated. Focusing again on the results obtained for high-generation dendrimers investigated with a large scattering vector q (or its modulus, more precisely) in order to probe the intramolecular dynamics, the plots of *S*(*q*, *t*) vs. *t* exhibited deviations from the simple exponential decay characteristic of the center-of-mass diffusion. As previously found [19], the exact $\Omega \left(q\right)/q^{3}$ (without pre-averaging the hydrodynamic approximation) plotted as a function of $qR_{g}$ exhibited a clear minimum for $qR_{g}\approx 2$, while pre-averaging led to a somewhat shallower minimum at $qR_{g}\approx 2.5$. Moreover, it was also shown that the good solvent expansion led to lower pre-averaging error compared to the phantom polymer with random-walk conformation, because the dendrimer expansion, even though still limited due to the topological connectivity constraint, somewhat relieves the density of the branch points that mostly affect the solvent flow. As for the comparison with the experimental data discussed previously [52], what was said when discussing La Ferla’s results still applies here, in view of the qualitative similarities of the theoretical results in this respect.

The viscoelastic complex modulus of semiflexible dendrimers was later investigated by Biswas’ group [32], adopting the same conformational model proposed in Reference [29] for the study of intrinsic viscosity. The low-frequency behavior had the usual power-law dependence presented in Equation (5), while the asymptotic behavior for ω → ∞ discussed in the paper ([G′] → ω^0^ and [G″] → ω^−1^) cannot have physical relevance; in fact, it would apply for ωτ*_N_* > 1, thus probing the internal dynamics of a spring at times shorter than the shortest relaxation time, and therefore beyond the validity of the adopted model (see again Figure 3). At intermediate frequencies, when probing the intramolecular dynamics in dendrimers with *g* > 4, a power-law dependence of [G′] and [G″] on ω was again recovered with an exponent close to 0.5 in particular for dendrimers were the local rigidity increased the distance from the branch point to the terminal beads. In particular, the semiflexible dendrimers at a generation *g* = 8 with a more open conformation due to the chosen orientation of the bond vectors across a branch point displayed exponents equal to α1 = 0.53 and α2 = 0.478 [32], which are the same as those found in Reference [24] for dendrimers with excluded-volume interactions.

The intramolecular dynamics of dendrimers were also investigated by Biswas et al. [61], accounting for the effect of the local excluded volume on nearest non-bonded beads belonging to the same and to the adjacent generation (see also Reference [31] for the intrinsic viscosity calculated with this model). Since the low-frequency behavior has a universal power-law dependence on ω as given by Equation (5) independently of the molecular topology and of the solvent quality, we now focus only on the intermediate frequency behavior relevant for the intramolecular dynamics. In this frequency range, a power-law dependence of [G′] and of [G″] on ω is again observed. The exponents of these power laws (see Equation (6)) were found to be in the range 0.574 < α1 < 0.522 and 0.485 < α2 < 0.432, with larger α values being found with greater excluded-volume parameters, and smaller values with smaller parameters. Note that, in this case, too, α1 is larger than α2, as found previously, while these values bracket those found for semiflexible dendrimers [32] and in the presence of a (non-local) excluded-volume expansion [24]. Accordingly, it may be stated that the local excluded-volume interactions may closely reproduce these different models, at least as far as the intramolecular mechanical behavior is concerned.

Biswas et al. also calculated the viscoelastic complex modulus of hyperbranched polymers with a random distribution of dead ends in consecutive shells through the conformational models described previously for the intrinsic viscosity. Thus, one paper dealt with the effect of the molecular rigidity [30], and another one with the effect of a local excluded-volume effect between bead pairs belonging to consecutive shells [31,61]. As before, only the intermediate frequency behavior, where the intramolecular dynamics are most relevant, is discussed. In the former model [30], a power-law dependence on ω was again recovered with a large total number of shells, with exponents equal to α1 = 0.487 and α2 = 0.521 in semiflexible hyperbranched molecules with a more expanded conformation, and to α1 = 0.587 and α2 = 0.632 in more compressed ones. On the other hand, in the latter model [31], the exponents describing the power-law dependence at intermediate ω in hyperbranched molecules with a large number of shells fell in the range 0.526 < α1 < 0.464 and 0.552 < α2 < 0.508. As mentioned previously for regular dendrimers, greater α values were found with larger excluded-volume parameters, and smaller ones with smaller parameters. However, it is noteworthy that in the hyperbranched polymers α2 > α1, that is, [G″] has a stronger dependence on ω than [G′], unlike what was shown for dendrimers; it is unclear, however, whether this different behavior is due to the presence of defects (the dead ends) per se, or to the slight changes in the hydrodynamic interactions they induce inside the molecule.

#### 3.2.2. Simulation Studies

To the best of our knowledge, the only simulation study of the viscoelastic complex modulus was performed by Freire et al. [48] using MC methods in continuous space, as first proposed in Reference [46], with the corrections described previously to account for the contribution of an individual friction bead with a finite size, for a realistic distribution of the angles formed by the bonds between adjacent branch points, and for a realistic distance distribution between adjacent branch points. This approach yielded a good agreement for the *g*-dependence of the intrinsic viscosity [46], and since it produced the relaxation times, it was then used to simulate the viscoelastic complex modulus measured under an oscillating shear deformation using Equations (2) and (3). In the intermediate frequency range, where the intramolecular dynamics are dominant, a power-law dependence of [G′] and [G″] on ω was clearly visible, which is consistent with Equation (6), in particular for the PAMAM dendrimers, possibly as a result of the larger number of flexible chemical bonds between consecutive branch points. Unfortunately, no explicit values for the power-law exponents were reported, even though the inequality α1 > α2 can be easily seen from the plots.

## 4. Conclusions and Outlook on Future Work

In this review, we describe the theoretical investigations into the dynamics of dendrimers in solution carried out with analytical theories and with molecular simulations. The most relevant observable quantity is undoubtedly the intrinsic viscosity [η], in view of its widespread and simple use in polymer characterization. A first important observation is that the familiar Mark-Houwink-Sakurada [η] = *KM^a^*, valid for linear, ring and star polymers, does not hold in the case of dendrimers. In fact, for the latter macromolecules, [η] does not increase monotonically with molar mass, as implied by Equation (4) with *a* > 0, but rather exhibits a maximum, occurring at some generation *g*. This behavior has been clearly shown in the case of PAMAM dendrimers [1,2,7], in Tri-PBzE dendrimers [8] and in Phosphorous-containing dendrimers [9], as well as in non-regular hyperbranched PAMAM molecules [10], but not in PPI dendrimers [11,12]. Clearly, the possibility remains that in the latter dendrimers, the experimental generations up to *g* = 5 (the numeration started from 1) were not large enough to display the maximum [η]. It is highly remarkable that these are the only systematic studies that investigate the dependence of [η] from the dendrimers generation and, to some extent, on the spacer length between adjacent branch points. Therefore, further experiments on higher-*g* PPI dendrimers and on further chemically different dendrimers would be most welcome to clarify this issue and to check whether the maximum [η] is indeed really a distinctive signature of the dendrimer topology irrespective of the local details.

Theoretical approaches to modeling the intrinsic viscosity do appear to show the same problem. In fact, while molecular simulations always predict a maximum [η] for some *g* value (even though quantitative discrepancies may sometimes be present), the predicted results of analytical theories are less clear, in a way. In fact, while this maximum was predicted with some specific conformational dendrimer models [29,33], with other models [19,20,25], this maximum was not predicted, at least for the number of generations taken into account. It is interesting, however, to stress that in Reference [29], the calculations of Reference [19] were extended to higher *g* values, producing a maximum [η], albeit not one in the correct position for most of the above-mentioned dendrimers. This result, and the trend in the calculated [η] values strongly suggest that in all cases, this maximum would indeed be present, albeit at a very high *g* and in a position not consistent with the experimental data.

There is a more general issue related with the conclusion of the previous paragraph. The intrinsic viscosity of dendrimers was investigated in analytical theories using two different viewpoints. The first one consists of applying the theoretical approach followed for linear (and star) polymers to dendrimers, obviously with the correct molecular topology [19,20,25]. The second one introduces some ad hoc assumptions that can be qualitatively justified in intramolecularly dense systems such as dendrimers [29,33], but which would not apply to linear chains. It could therefore be argued that a full, consistent theory that can be applied to polymers of any topology and is in good agreement with experiments is still lacking. In the same spirit, we also point out that the approach of Lu et al. [34,35], which we described as a mesoscopic model of polymers, appears to be satisfactory for all of the above topologies, but in our opinion it lacks a sound microscopic model, such that in this case, too, further analysis would be welcome.

Other experiments are able to probe more directly the intramolecular dynamics of dendrimers, in particular at the so-called segmental scale. These experiments can probe the mechanical behavior under applied oscillatory stress through the viscoelastic complex modulus in terms of [G′] and [G″], that is, the storage and the loss modules, as a function of the applied frequency ω. In this connection, it should be pointed out, however, that certain approaches did not show any maximum as a function of *g* for intrinsic viscosity, for instance, Reference [20], unlike other studies that did so, such as [29], while the results obtained for the viscoelastic complex modulus were qualitatively quite similar, being different only in the details. Accordingly, this experimental technique appears to depend on topology, but does not allow for clear discrimination between different theories. Another experiment probing the segmental dynamics, or more precisely the intramolecular correlation among the beads at different times, consists of quasi-elastic scattering, measuring the dynamic structure factor. Unfortunately, very few experimental data of this quantity are available for dendrimers in solution, unlike what can be found for linear and, at least in part, for star polymers. At the same time, only a few theoretical investigations have explicitly considered this quantity (apart from References [19,24]), and even fewer through experimental studies (see Reference [53]), where these could provide important information. We thus believe that more experiments and modeling studies of the dynamic structure factor of dendrimers and of highly branched polymers would be most useful.

We feel that future experimental and theoretical work on these issues will eventually provide useful information for tuning the behavior of dendrimers, either in themselves or after suitable functionalization of the end units or, in some cases, also in their interior parts, for a wide range of applications ranging from drug delivery systems [3] to nucleic-acid transfection system [5] and theranostics [6] or nanomedicine, catalysis and molecular electronics [4]. The still growing interest in these highly branched molecules is also evident in most recent extensive reviews discussing, for instance dendrimer-based nanoparticles for cancer treatment as drug carriers through non-covalent encapsulation or bound to the functionalized ends [62], or dendrimers acting as electrochemical biosensors and sensors through functionalization of the free ends [63], and dendrimers having functionalized ends for the removal of contaminants in water environment [64]. It is remarkable that in these applications, the dendrimer dynamics is relevant for the release of the encapsulated molecules, or for an efficient functionalization of the free ends that must be mostly found at the molecular envelope in order to achieve their maximum efficiency.
